# Screening Deep Eutectic Solvents for CO_2_ Capture With COSMO-RS

**DOI:** 10.3389/fchem.2020.00082

**Published:** 2020-02-13

**Authors:** Yanrong Liu, Hang Yu, Yunhao Sun, Shaojuan Zeng, Xiangping Zhang, Yi Nie, Suojiang Zhang, Xiaoyan Ji

**Affiliations:** ^1^Energy Engineering, Division of Energy Science, Luleå University of Technology, Luleå, Sweden; ^2^State Key Laboratory of Material-Oriented Chemical Engineering, Nanjing Tech University, Nanjing, China; ^3^CAS Key Laboratory of Green Process and Engineering, Beijing Key Laboratory of Ionic Liquids Clean Process, State Key Laboratory of Multiphase Complex Systems, Institute of Process Engineering, Chinese Academy of Sciences, Beijing, China; ^4^Zhengzhou Institute of Emerging Industrial Technology, Zhengzhou, China; ^5^School of Chemical Engineering, University of Chinese Academy of Sciences, Beijing, China

**Keywords:** deep eutectic solvents, CO_2_ capture, COSMO-RS, CO_2_ solubility, Henry's constant

## Abstract

In this work, 502 experimental data for CO_2_ solubilities and 132 for Henry's constants of CO_2_ in DESs were comprehensively summarized from literatures and used for further verification and development of COSMO-RS. Large systematic deviations of 62. 2, 59.6, 63.0, and 59.1% for the logarithmic CO_2_ solubilities in the DESs (1:2, 1:3, 1:4, 1:5), respectively, were observed for the prediction with the original COSMO-RS, while the predicted Henry's constants of CO_2_ in the DESs (1:1.5, 1:2, 1:3, 1:4, 1:5) at temperatures ranging of 293.15–333.15 K are more accurate than the predicted CO_2_ solubility with the original COSMO-RS. To improve the performance of COSMO-RS, 502 data points of CO_2_ solubility in the DESs (1:2, 1:3, 1:4, 1:5) were used for correcting COSMO-RS with a temperature-pressure dependent parameter, and the CO_2_ solubility in the DES (1:6) was predicted to further verify the performance of the corrected model. The results indicate that the corrected COSMO-RS can significantly improve the model performance with the ARDs decreasing down to 6.5, 4.8, 6.5, and 4.5% for the DESs (1:2, 1:3, 1:4, and 1:5), respectively, and the corrected COSMO-RS with the universal parameters can be used to predict the CO_2_ solubility in DESs with different mole ratios, for example, for the DES (1:6), the corrected COSMO-RS significantly improves the prediction with an ARD of 10.3% that is much lower than 78.2% provided by the original COSMO-RS. Additionally, the result from COSMO-RS shows that the σ-profiles can reflect the strength of molecular interactions between an HBA (or HBD) and CO_2_, determining the CO_2_ solubility, and the dominant interactions for CO_2_ capture in DESs are the H-bond and Van der Waals force, followed by the misfit based on the analysis of the predicted excess enthalpies.

**Graphical Abstract:**
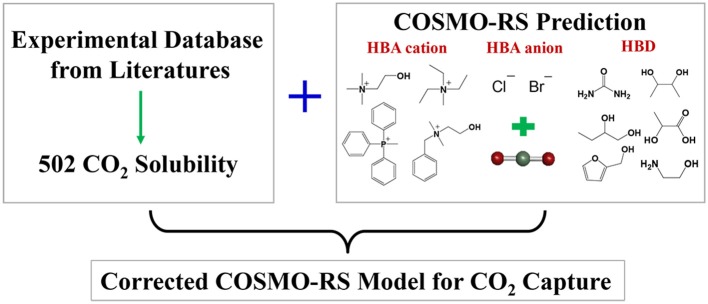
Corrected COSMO-RS model for screening CO_2_ solubility in DES.

## Introduction

With the rapid development of industry, the demand for energy is growing. Fossil fuels currently account for the majority of energy supply (Rahmanifard and Plaksina, 2019). The use of fossil fuels poses a range of environmental problems. For example, the use of fossil fuels emits a large amount of carbon dioxide (CO_2_) (Hanif et al., 2019; Hosseini et al., 2019). This, in turn, leads to the serious greenhouse-gas effect. It was reported that the global CO_2_ emissions reached an all-time high of 33.143 billion tons in 2018 by the International Energy Agency (IEA) (Qu, 2018). This calls for CO_2_ capture.

There are several technologies for CO_2_ capture, for example, physical, or chemical solvent scrubbing (Abdeen et al., 2016) and pressure swing adsorption (Riboldi and Bolland, 2016). Because of the complexity of the gas components, most technologies still suffer from high energy demand, high cost, and serious secondary pollutions. Developing new capture technologies, including new solvents and novel processes, is the key point to CO_2_ capture.

Recently, ionic liquids (ILs) have been proposed as potential candidates for CO_2_ capture due to their unique properties. However, the conventional ILs are expensive mainly due to the complex synthesis process. The newly emerged DESs possess analogous properties to ILs and share many common properties with ILs, whereas DESs have additional merits of low cost, biodegradability, low toxicity, easy preparation, and no purification requirement (Sarmad et al., 2017a). The most fascinating property of DESs is the structural diversity, and it can be prepared by mixing a hydrogen bond donor (HBD) with a hydrogen bond acceptor (HBA) in appropriate mole ratios (Hayyan et al., 2013).

To develop DESs for CO_2_ capture, the CO_2_ absorption capacity (i.e., CO_2_ solubility) in DESs is one of the important properties. It is reported that the CO_2_ solubility in DESs depends on pressure, temperature, and the mole ratio of HBD to HBA, and it increases with increasing pressure and decreasing temperature (Sarmad et al., 2017a). Based on the studies of Kamps et al. (2003) and Aki et al. (2004), it was found that the CO_2_ solubilities in DESs were comparable with the imidazolium-based ILs. Leron and Li measured the CO_2_ solubilities in choline chloride (ChCl)-glycerol (GLY) 1:2 at 313.15 K and 0–6 MPa (Leron and Li, 2013). The result indicated that the measured CO_2_ solubilities (0.1103–3.0718 mol/kg) in this DES are greater than those in the 1-ethyl-3-methylimidazolium-based ILs, such as 1-ethyl-3-methylimidazolium tetrafluoroborate, 1-ethyl-3-methylimidazolium dicyanamide, and 1-ethyl-3-methylimidazolium ethyl sulfate. In the work by Sarmad et al., 35 DESs with 209 data points for CO_2_ solubility at 298.15 K and below 2 MPa were reported (Sarmad et al., 2017b). Among the synthesized DESs, 15 samples exhibit higher CO_2_ solubilities than the conventional ILs, such as 1-octyl-3-methylimidazolium tetrafluoroborate, 1-octyl-3-methylimidazolium hexafluorophosphate, and 1-ethyl-3-methylimidazolium ethylsulfate. Sarmad et al. reviewed the CO_2_ solubilities in 45 DESs in wide ranges of mole ratio (HBA:HBD), temperature, and pressure (Sarmad et al., 2017a). Based on this review, top three DESs with respect to CO_2_ solubilities were acquired, i.e., 3.6929, 3.5592, and 3.1265 mol/kg for ChCl-GLY (1:2, 303.15 K, 5.863 MPa), ChCl-Urea (1:2, 303.15 K, 5.654 MPa), and ChCl-ethylene glycerol (EG) (1:2, 303.15 K, 0.5774 MPa), respectively.

Numerous possible DESs can be synthesized. It is nevertheless a challenge to identify and suggest the best DESs for capturing CO_2_ based on the available experimental measurements only. A rapid and priori screening method to predict the CO_2_ absorption capacity in DESs is needed. COSMO-RS (Conductor-like Screening Model for Real Solvents) is recommended for predicting some thermodynamic properties (Gonzalez-Miquel et al., 2011; Liu Y.-R., et al., 2016), such as activity coefficients, solubilities, and Henry's constants, and it can also be used for calculating the properties of mixtures at various temperatures and pressures, rendering it an effective tool to predict and develop task-specific DESs for a specific application. Previous work has demonstrated that Henry's constants can be used as one of the criteria to screen ILs for CO_2_ capture (Manan et al., 2009; Palomar et al., 2011), and the Henry's constants of CO_2_ in ILs have been successfully estimated with COSMO-RS (Gonzalez-Miquel et al., 2011). However, using COSMO-RS to predict the CO_2_ solubility or the Henry's constant of CO_2_ in DESs is still scarce. To the best of our knowledge, only Kamgar et al. (2017b) predicted CO_2_ solubility in the DES of ChCl-Urea (1:2) with COSMO-RS, demonstrating a reliable prediction only at low pressures and high temperatures, where the gas could be assumed to be an ideal gas. No report is available to predict the Henry's constants of CO_2_ in DESs and compare with the experimental results for verification.

The aim of this work was to predict CO_2_ solubilities, the Henry's constants of CO_2_, and the interactions for CO_2_ capture in DESs with COSMO-RS. A comprehensive survey of the published experimental results of CO_2_ solubility and Henry's constant of CO_2_ was firstly carried out. COSMO-RS was used to predict these two properties, and the predictions were compared with the experimental results. COSMO-RS was further developed with a correction based on the experimental CO_2_ solubility to improve the model performance. The σ-profiles predicted by COSMO-RS were used to reflect the strength of molecular interactions between an HBA (or HBD) and CO_2_, and the calculated excess enthalpy was applied to acquire the dominant interactions for CO_2_ capture in DESs.

## Computational Detail

### COSMO-RS Computation Details

COSMO-RS calculations were performed using the software COSMOtherm (version C3.0, release 14.01, applied with parameterization BP_TZVP_C30_1401, COSMOlogic, Leverkusen, Germany) (Larriba et al., 2017). Following the standard method, first, the quantum chemical Gaussian09 package was used to optimize the structure of the studied compounds at the B3LYP/6-31++G (d, p) level. Second, the COSMOfiles of the optimized structures were opened by Gaussian03, and the COSMO continuum solution models were obtained using the BVP86/TZVP/DGA1 level theory. Third, CO_2_ solubility, the Henry's constant of CO_2_, σ-profiles, and excess enthalpy were determined with COSMO-RS (Liu Y.-R., et al., 2016). In computation, the temperature and pressure were set to be the same values as the experimental conditions.

In COSMO-RS, all DESs were implemented in COSMOtherm software following the electroneutral approach, where each DES was treated as three different compounds in a stoichiometric mixture (Larriba et al., 2017). For HBA, the mole fractions of the cation and anion were treated as equal, i.e., *n*_cation_ = *n*_anion_ = *n*_HBA_. The solubility and Henry's constant of CO_2_ were calculated based on the equations described by Klamt et al. (2001) and Loschen and Klamt (2014). The total and contribution of the excess enthalpies were calculated with the method reported by Casas et al. (2012).

In COSMO-RS, the solubility (*X*) (i.e., the mole fraction) of CO_2_ in DESs was obtained with the following equation (Equation 1) (Li et al., 2014).

(1) $$\begin{array}{l} X_{{CO}_{2}}=\frac{n_{{CO}_{2}}}{n_{{CO}_{2}}+n_{DES}} \end{array}$$

where *n*_*DES*_ is the mole quantity of absorbent, and it was obtained according to the mass weight and the mole mass of DESs.

To evaluate the model performance, the discrepancies between the results (i.e., CO_2_ solubility *X*, Henry's constant *H*) estimated with COSMO-RS and the corresponding experimental data points were quantified with the absolute relative error (ARD) as defined by Equation 2 (Kamgar et al., 2017a).

(2) $$\begin{array}{l} \text{ARD}\%=\frac{100}{NP}\sum_{i=1}^{NP}\left|\frac{Y^{Exp.}-Y^{\text{COSMO}-\text{RS}}}{Y^{Exp.}}\right| \end{array}$$

where *NP* is the total number of data points. *Y*^*COSMO*−*RS*^ (i.e., $\text{ln }{\text{X}}_{\text{C}{\text{O}}_{2}}^{\text{COSMO}-\text{RS}}$ or H^COSMO−RS^) is the result predicated with COSMO-RS (original or corrected) at a given temperature and pressure, and *Y*^*Exp*.^ is the corresponding experimental result (i.e., $\text{ln }X_{CO_{2}}^{Exp.}$ or *H*^*Exp*.^).

### DESs-Database and COSMOfiles

A literature survey was conducted, and the DESs with CO_2_ solubility and the Henry's constant of CO_2_ in DESs were summarized and used as databases for verifying and further developing COSMO-RS. According to the survey, for CO_2_ solubility, the DESs with HBA:HBD at the mole ratios of 1:2, 1:3, 1:4, and 1:5 have more experimental results compared to those with other mole ratios, and they were selected for developing COSMO-RS model in this work, while the limited experimental results at the mole ratio of 1:6 were used for verification of the developed COSMO-RS. For Henry's constants, the DESs (1:1.5, 1:2, 1:3, 1:4, 1:5) were chosen due to that sufficient experimental results are available for these DESs in literatures. The DESs databases together with the experimental measurement conditions are summarized in Tables 1, 2, respectively, and the detailed CO_2_ solubilities and the Henry's constants under different conditions are provided in Tables S1–S6.

**Table 1:** DESs database studied in this work for CO_2_ solubility.

**DES**	**HBA**	**HBD**	**Mole ratio (HBA:HBD)**	**Temperature range (K)**	**Press range (KPa)**	**Numbers of data**	**References**
ChCl-Phenol	Choline chloride	Phenol	1:2	293.15–323.15	99–520.2	19	Li et al., 2014
BTEACl-AC	Benzyltriethylammonium chloride	Acetic acid	1:2	298.15	325–2054	6	Sarmad et al., 2017b
BTMACl-AC	Benzyltrimethylammonium chloride	Acetic acid	1:2	298.15	219–2037	7	
BTMACl-GLY	Benzyltrimethylammonium chloride	Glycerol	1:2	298.15	394–2026	6	
TBACl-AC	Tetrabutylammonium chloride	Acetic acid	1:2	298.15	348–2002	6	
TBABr-AC	Tetrabutylammonium bromide	Acetic acid	1:2	298.15	388–2011	5	
TEACl-AC	Tetraethylammonium chloride	Acetic acid	1:2	298.15	281–2018	6	
TEMACl-AC	Triethylmethylammonium chloride	Acetic acid	1:2	298.15	198–1837	7	
TEMACl-EG	Triethylmethylammonium chloride	Ethylene glycol	1:2	298.15	138–1345	6	
TEMACl-LA	Triethylmethylammonium chloride	Lactic acid	1:2	298.15	143–1863	6	
TEMACl-LEV	Triethylmethylammonium chloride	Levulinic acid	1:2	298.15	136–1617	5	
TEMACl-GLY	Triethylmethylammonium chloride	Glycerol	1:2	298.15	150-1648	5	
TBACl-LA	Tetrabutylammonium chloride	Lactic acid	1:2	308, 318	93–1992, 93–1992	28	Zubeir et al., 2014
TEACl-LA	Tetraethylammonium chloride	Lactic acid	1:2	308, 318	97–1993, 94–1992	40	
TMACl-LA	Tetramethylammonium chloride	Lactic acid	1:2	308, 318	98–1992, 95–1993	40	
ChCl-DEG	Choline chloride	Diethylene glycol	1:3	293.15–323.15	112.8–524	20	Li et al., 2014
ChCl-FA	Choline chloride	Furfuryl alcohol	1:3	303.15–333.15	80.9–586.4	24	Lu et al., 2015
ChCl-LEV	Choline chloride	Levulinic acid	1:3	303.15–333.15	69.8–579.8	24	
ChCl-Phenol	Choline chloride	Phenol	1:3	293.15–323.15	104.4–514.4	20	
ChCl-TEG	Choline chloride	Triethylene glycol	1:3	293.15–323.15	109.3–516	20	Li et al., 2014
TEACl-AC	Tetraethylammonium chloride	Acetic acid	1:3	298.15	397–2016	6	Sarmad et al., 2017b
TEACl-OCT	Tetraethylammonium chloride	Octanoic acid	1:3	298.15	353–2018	6	
MTPPBr-LEV	Methyltriphenyl phosphonium bromide	Levulinic acid	1:3	298.15	301–2068	7	
ChCl-DEG	Choline chloride	Diethylene glycol	1:4	293.15–323.15	110.4–526.9	20	Li et al., 2014
ChCl-FA	Choline chloride	Furfuryl alcohol	1:4	303.15–333.15	65.2–585.4	24	Lu et al., 2015
ChCl-LEV	Choline chloride	Levulinic acid	1:4	303.15–333.15	60–587.4	24	
ChCl-Phenol	Choline chloride	Phenol	1:4	293.15–323.15	108.2–529.1	20	Li et al., 2014
ChCl-TEG	Choline chloride	Triethylene glycol	1:4	293.15–323.15	109.3–520.3	20	
TMACl-AC	Tetramethylammonium chloride	Acetic acid	1:4	298.15	294–1741	6	Sarmad et al., 2017b
TPACl-EA	Tetrapropylammonium chloride	Ethanolamine	1:4	298.15	481–2009	6	
MTPPBr-AC	Methyltriphenyl phosphonium bromide	Acetic acid	1:4	298.15	173–2014	8	
MTPPBr-1,2-PRO	Methyltriphenyl phosphonium bromide	1,2-Propanediol	1:4	298.15	220–2026	7	
ChCl-LEV	Choline chloride	Levulinic acid	1:5	303.15–333.15	71.5–581	24	Lu et al., 2015
ChCl-FA	Choline chloride	Furfuryl alcohol	1:5	303.15–333.15	70.9–577.2	24	
TPACl-AC	Tetrapropylammonium chloride	Acetic acid	1:6	298.15	350–2030	6	Sarmad et al., 2017b

**Table 2:** DESs database studied in this work for Henry's constants of CO_2_.

**DES**	**HBA**	**HBD**	**Mole ratio (HBA:HBD)**	**Temperature range (K)**	**Points**	**References**
ChCl-Urea	Choline chloride	Urea	1:1.5	313.15–333.15	3	Li et al., 2008
ChCl-Urea	Choline chloride	Urea	1:2	313.15–333.15	3	
ChCl-Urea	Choline chloride	Urea	1:2	308.2–328.2	3	Xie et al., 2013
ChCl-Phenol	Choline chloride	Phenol	1:2	293.15–323.15	4	Li et al., 2014
TMACl-LA	Tetramethylammonium chloride	Lactic acid	1:2	308–318	2	Zubeir et al., 2014
TEACl-LA	Tetraethylammonium chloride	Lactic acid	1:2	308–318	2	
TBACl-LA	Tetrabutylammonium chloride	Lactic acid	1:2	308–318	2	
TBACl-DecA	Tetrabutylammonium chloride	Decanoic acid	1:2	298.15–323.15	3	Zubeir et al., 2018
N_8881_Br-DecA	Methyltrioctylammonium bromide	Decanoic acid	1:2	298.15–323.15	3	
N_8881_Cl-DecA	Methyltrioctylammonium bromide	Decanoic acid	1:2	298.15–308.15	2	
ChCl-EG	Choline chloride	Ethylene glycol	1:2	303.15	1	Haider et al., 2018
TBABr-EG	Tetrabutyl ammonium bromide	Ethylene glycol	1:2, 1:3, 1:4	303.15	1, 1, 1	
TBABr-DEG	Tetrabutyl ammonium bromide	Diethylene glycol	1:2, 1:3, 1:4	303.15	1, 1, 1	
ChCl-1,2 PRO	Choline chloride	1,2-propanediol	1:3, 1:4	293.15–323.15	4, 4	Chen et al., 2014
ChCl-2,3 BUT	Choline chloride	2,3-butanediol	1:3, 1:4	293.15–323.15	4, 4	
ChCl-1,4 BUT	Choline chloride	1,4-butanediol	1:3, 1:4	293.15–323.15	4, 4	
ChCl-DEG	Choline chloride	Diethylene glycol	1:3, 1:4	293.15–323.15	4, 4	Li et al., 2014
ChCl-FA	Choline chloride	Furfuryl alcohol	1:3, 1:4	293.15–323.15	4, 4	Lu et al., 2015
ChCl-LEV	Choline chloride	Levulinic acid	1:3, 1:4	293.15–323.15	4, 4	
ChCl-Phenol	Choline chloride	Phenol	1:3, 1:4	293.15–323.15	4, 4	Li et al., 2014
ChCl-TEG	Choline chloride	Triethylene glycol	1:3, 1:4	293.15–323.15	4, 4	
ChCl-GC	Choline chloride	Guaiacol	1:3, 1:4, 1:5	293.15–323.15	4, 4, 4	Liu X. B. et al., 2017
ACCl-GC	Acetylcholine chloride	Guaiacol	1:3, 1:4, 1:5	293.15–323.15	4, 4, 4	
ATPPBr-DEG	Allyltriphenyl phosphonium bromide	Diethylene glycol	1:4	303.15	1	Ghaedi et al., 2017
ATPPBr-TEG	Allyltriphenyl phosphonium bromide	Triethylene glycol	1:4	303.15	1	
ChCl-FA	Choline chloride	Furfuryl alcohol	1:5	303.15–333.15	4	Lu et al., 2015
ChCl-LEV	Choline chloride	Levulinic acid	1:5	303.15–333.15	4	

To use COSMO-RS, the COSMOfiles for all the studied HBAs and HBDs are needed. In this work, those for the HBAs of ATPP^+^, AC^+^, BTEA^+^, BTMA^+^, and MTPP^+^, and for the HBDs of lactic acid (LA), ethylene glycol (EG), levulinic acid (LEV), furfuryl alcohol (FA), triethylene glycol (TEG), guaiacol (GC), decanoic acid (DecA), 1,4-butanediol (1,4-BUT), and 2,3-butanediol (2,3-BUT) were calculated based on the procedures described in the computation details. The optimized structures of HBAs and HBDs as well as the COSMOfiles from Gaussian are provided in Supplementary Information. The COSMOfiles for other HBAs and HBDs studied in this work as listed in Tables 1, 2 were directly taken from the COSMO-RS database.

## Model Results and Discussion

### CO_2_ Solubility With COSMO-RS

For conducting COSMO-RS prediction, 502 experimental data points of CO_2_ solubilities in four types of DESs (1:2, 1:3, 1:4, 1:5) under different conditions summarized from the published work were used as the input to predict the CO_2_ solubility. The model predictions were then compared with the experimental results. Together with the selected 502 experimental data points of CO_2_ solubilities, the predicted results and the corresponding ARDs are listed in Tables S1–S4.

Taking the results listed in Table S1 as examples, it can be observed that COSMO-RS is capable of predicting CO_2_ solubilities in DESs, but qualitatively. For example, the experimental ln *X*_*CO*_2__ of TMACl-LA (1:2) is −5.1743 at 308 K, 194 kPa, and −5.2419 at 318 K, 194 kPa. The increase of temperature from 308 to 318 K leads to a decrease of CO_2_ solubility with a difference of (Δln *X*_*CO*_2__ = −0.0676). The corresponding COSMO-RS predicted values of ln *X*_*CO*_2__ are −2.9257 and −3.1451, respectively, indicating a temperature increase results in a decreased CO_2_ solubility, however, the difference is (Δln *X*_*CO*_2__ = −0.2194) that are much lower than the experimental observation. For different kinds of DESs, the predicted CO_2_ solubility also provides the same trend with the experiment results, for example, the experimental ln *X*_*CO*_2__ for TBACl-LA (1:2) is −4.3513 at 308 K, 194 kPa, which is greater than that of TMACl-LA (1:2) under the same condition. The prediction of COSMO-RS provides a value of ln *X*_*CO*_2__ = −2.2662 for TBACl-LA (1:2) at 308 K, 194 kPa, which is also higher than that for TMACl-LA (1:2) (i.e., ln *X*_*CO*_2__ = −2.9257). The results for other DESs listed in Tables S2–S4 show similar observations as those listed in Table S1. Hence, COSMO-RS can be used to screen DESs qualitatively, reducing the amount of unnecessary experimental measurements.

**Figure 1:**
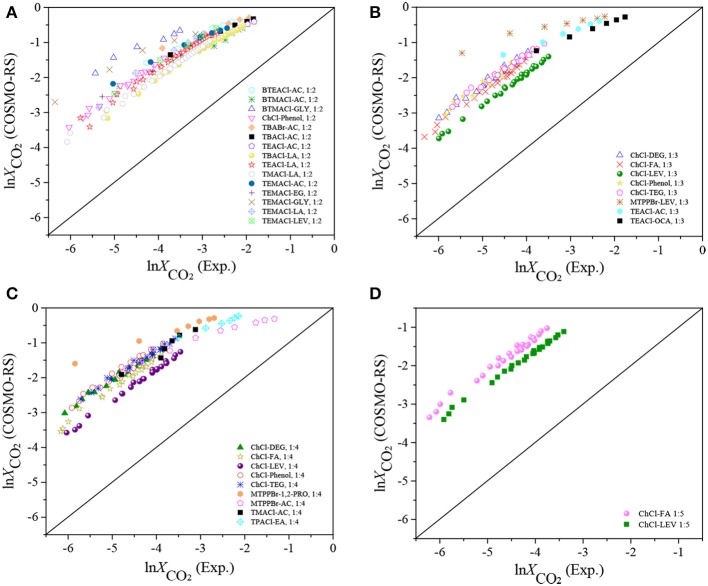
Experimental ln *X*_*CO*_2__ vs. the ln *X*_*CO*_2__ predicted with the original COSMO-RS. **(A)** HBA:HBD 1:2; **(B)** HBA:HBD 1:3; **(C)** HBA:HBD 1:4; **(D)** HBA:HBD 1:5.

Further comparisons were performed based on ln *X*_*CO*_2__ between the experimental and COSMO-RS results. As shown in Figure 1, systematic deviations can be observed, i.e., all the COSMO-RS predictions are higher than the experimental results. With increasing pressure and decreasing temperature, the discrepancies become larger, which is consistent with the observation by Kamgar et al. (2017b). This indicates that using the COSMO-RS with the parameters obtained from the conventional compound systems to predict the thermodynamic properties of DESs will lead to large deviations, which was also pointed out by others (Han et al., 2018). One of the reasons can be attributed to the formation of nanoscopic structures in DESs (Cerajewski et al., 2018), which is completely different from the solvation properties of molecular solutes in the conventional solvents at the microscopic scale.

### Henry's Constant With COSMO-RS

**Figure 2:**
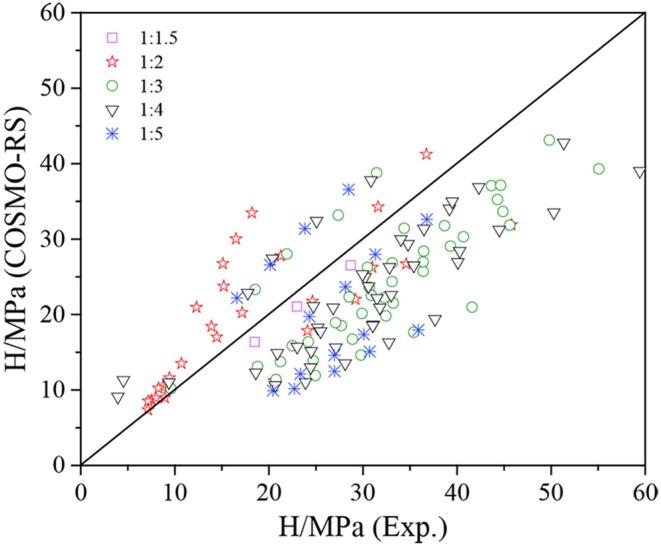
Experimental vs. predicted Henry's constants with the original COSMO-RS.

The Henry's constants of CO_2_ in DESs (1:1.5, 1:2, 1:3, 1:4, 1:5) with 132 data points were predicated with COSMO-RS and compared with the experimental results (Figure 2). The specific values of the experimental and predicted Henry's constants are listed in Table S6, and the ARDs are summarized in Table 3. From Table S6, it can be found that the predicted Henry's constants are in agreement with the experimental data, and they increase with increasing temperature. As shown in Figure 2, most of the predicted Henry's constants are lower than the experimental values, which is consistent with the observation for the CO_2_ solubility because the Henry's constant is inversely proportional to the CO_2_ solubility (Liu X. Y., et al., 2016). A 13.7–36.3% deviation can be observed as listed in Table 3, indicating that the predicated Henry's constants are more accurate than the predicted CO_2_ solubility with the original COSMO-RS (Table 4).

**Table 3:** ARDs for the Henry's constants determined experimentally with respect to those predicted with the original COSMO-RS.

HBA:HBD	1:1.5	1:2	1:3	1:4	1:5
ARD	13.7%	29.9%	28.6%	34.4%	36.3%

**Table 4:** Corrected COSMO-RS for predicting ln *X*_*CO*_2__ in the DESs of four mole ratios.

**Mole ratio**	**Correction, T in K, P in KPa $\text{ln}\;{\text{X}}_{{\text{CO}}_{2}}^{\text{corr}.}=\text{ln }{\text{X}}_{{\text{CO}}_{2}}^{\text{COSMO}-\text{RS}}-\text{A}\times \frac{1}{\text{T}}+\text{BP}+\text{C}$**	**ARDs compared to experimental ln X_CO_2__**	
		**Before correction**	**After correction**
1:2	(3) $\text{ln }X_{CO_{2}}^{Corr.}=\text{ln }X_{CO_{2}}^{COSMO-RS}-1.1980\times 10^{3}\frac{1}{T}+3.4453\times 10^{-4}P+1.4186\;$	62.2%	6.5%
1:3	(4) $\text{ln }X_{CO_{2}}^{Corr.}=\text{ln }X_{CO_{2}}^{COSMO-RS}-9.4659\times 10^{2}\frac{1}{T}+5.4618\times 10^{-4}P+0.2488$	59.6%	4.8%
1:4	(5) $\text{ln }X_{CO_{2}}^{Corr.}=\text{ln }X_{CO_{2}}^{COSMO-RS}-8.6186\times 10^{2}\frac{1}{T}+6.4121\times 10^{-4}P-0.1682$	63.0%	6.5%
1:5	(6) $\text{ln }X_{CO_{2}}^{Corr.}=\text{ln }X_{CO_{2}}^{COSMO-RS}-2.2721\times 10^{2}\frac{1}{T}+5.1644\times 10^{-4}P-2.0832$	59.1%	4.5%

### COSMO-RS Correction for CO_2_ Solubility

**Figure 3:**
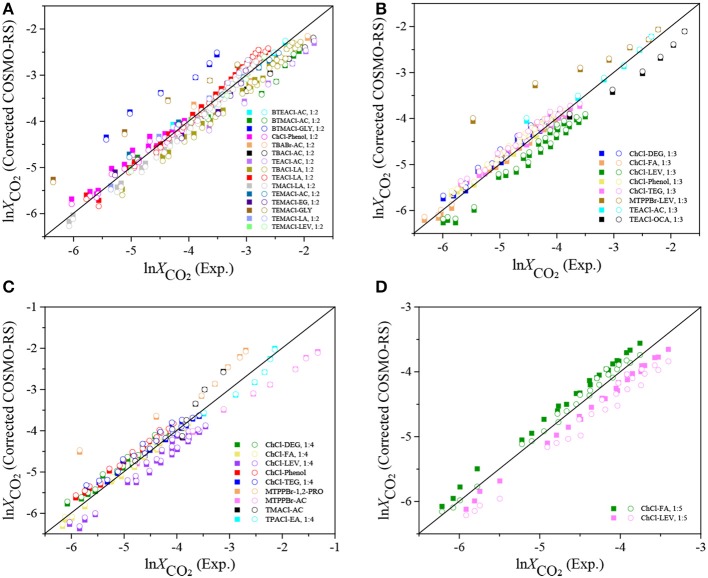
Experimental ln *X*_*CO*_2__ vs. the ln *X*_*CO*_2__ calculated with the corrected COSMO-RS models using Equations 3–6 (square symbol) and Equation 10 (circular symbol). **(A)** HBA:HBD 1:2; **(B)** HBA:HBD 1:3; **(C)** HBA:HBD 1:4; **(D)** HBA:HBD 1:5.

Although, from one side, it is unfortunate to observe a large deviation between the experimental CO_2_ solubility and the COSMO-RS prediction, from the other side, it illustrates that it is possible to develop a systematic correction of COSMO-RS for improving the model performance of CO_2_ solubility in DESs. Liu et al. reported a corrected COSMO-RS for predicting the activity coefficient of CO_2_ in ILs and acquired a good agreement between the experimental and predicted results after correction (Liu et al., 2018). Following this idea, in this work, a temperature-pressure-dependent correction was firstly proposed as summarized in Equations 3–6 (Table 4) for the DESs with the same mole ratio. As displayed in Table 4, the corrected COSMO-RS includes three parameters, i.e., *A* (K^−1^), *B* (KPa^−1^), and *C*, and these parameters were adjusted based on the experimental CO_2_ solubility at different temperatures and pressures for each group of DESs, i.e., the DESs with the same mole ratio. The predicted results with the corrected COSMO-RS are given in Tables S1–S4. The performance of the corrected COSMO-RS is further illustrated in Figure 3, and the deviations in ARDs between the experimental and modeling results are reported in Table 4. It can be found that, with the corrected COSMO-RS, i.e., the COSMO-RS with a temperature-pressure-dependent parameter, the predicted logarithmic CO_2_ solubilities (square symbol in Figure 3) are in agreement with the experimental results, with much smaller ARDs of 6.5, 4.8, 6.5, and 4.5% for these four groups of DESs compared to the ARDs with the original COSMO-RS. According to the results listed in Tables S1–S4, the ARD decreases with increasing temperature, which agrees with the observation by Kamgar et al. (2017b). As shown in Table 4, the adjustable parameters *A* (K), *B* (KPa^−1^), and *C* in four corrected Equations 3–6 are almost linearized with the mole ratios. To further improve the model prediction capacity, the adjustable parameters *A, B*, and *C* were set to be mole-ratio-dependent which can be described as follows:

(7) $$\begin{array}{l} A=k_{1}\times w+k_{2} \end{array}$$

(8) $$\begin{array}{l} B=k_{3}\times w+k_{4} \end{array}$$

(9) $$\begin{array}{l} C=k_{5}\times w+k_{6} \end{array}$$

where *w* is the mole ratio, being 2, 3, 4, 5, etc.

To obtain *k*_1_-*k*_6_, in this work, the experimental results of CO_2_ solubilities in the DESs with four mole-ratios of 1:2, 1:3, 1:4, and 1:5 were used, and, in total, 502 experimental data points were included in fitting with a linear least square method. The fitted parameters of *k*_1_-*k*_6_ together with the corresponding ARDs are listed in Table 5, and the fitted CO_2_ solubilities are given in Tables S1–S4.

**Table 5:** Adjustable parameters of Equation 10 and the corresponding ARDs.

**Adjustable parameters**						**ARDs compared to experimental ln X_CO_2__**			
*k*_1_/(K)	*k*_2_/(K)	*k*_3_/(KPa^−1^)	*k*_4_/(KPa^−1^)	*k*_5_	*k*_6_	1:2	1:3	1:4	1:5
48.5344	−1094.7706	9.4696 × 10^−5^	2.1975 × 10^−4^	−0.3249	1.3164	6.8%	5.2%	6.6%	4.7%

**Figure 4:**
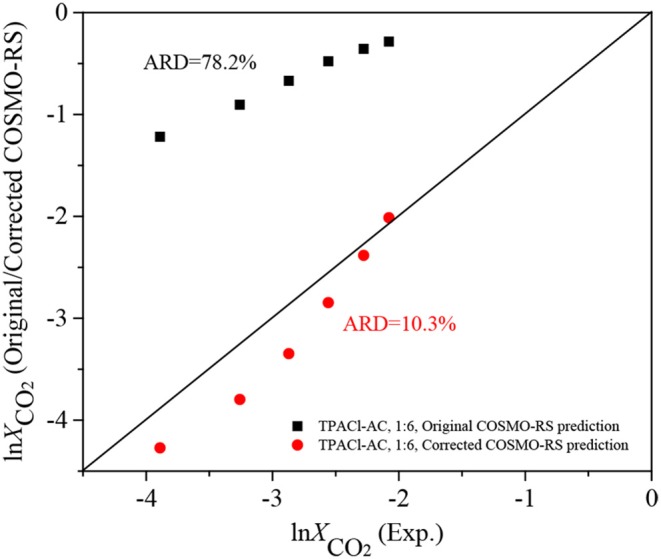
Experimental ln *X*_*CO*_2__ vs. the ln *X*_*CO*_2__ predicted with the original and corrected COSMO-RS (Equation 10) at the mole ratio of 1:6.

To illustrate the fitting performance with this set of universal parameters (*k*_1_-*k*_6_), the CO_2_ solubilities obtained with the corrected COSMO-RS (circular symbol) were compared with the experimental results as well as those with the corrected COSMO-RS but using the individual parameters at each mole-ratio. As shown in Figure 4, the predicted $\text{ln }X_{CO_{2}}^{corr.}$ with the corrected COSMO-RS using the universal parameters (i.e., Equation 10) agrees with the experimental data, and only a slight deviation can be observed between these two corrected COSMO-RS models, i.e., with Equation 10 and Equations 3–6, respectively. Additionally, from Table 5, it can be found that the ARDs with Equation 10 are 6.8, 5.2, 6.6, and 4.7% for the DESs at mole ratios of 1:2, 1:3, 1:4, and 1:5, respectively, which are almost the same as the ARDs calculated individually with Equations 3–6 (Table 4). All these indicate that it is reasonable to use the mole-ratio-dependent parameters.

(10) $$\begin{array}{l} \text{ln }X_{CO_{2}}^{corr.}=\text{ln }X_{CO_{2}}^{COSMO-RS}+\left(k_{1}\times w+k_{2}\right)\times \frac{1}{T}+\left(k_{3}\times w+k_{4}\right)\\ \;\times P+\left(k_{5}\times w+k_{6}\right) \end{array}$$

### Further Verification of the Corrected COSMO-RS

COSMO-RS with the universal parameters *k*_1_–*k*_6_ can be used to predict CO_2_ solubilities in the DESs with any mole ratios according to Equation 10_._ In order to further investigate the model prediction capacity, it was used to predict CO_2_ solubilities in the DESs at the mole ratio of 1:6. This type of DES was excluded in parameter fitting for correcting COSMO-RS due to the limited number of available experimental data points. The comparison of the prediction with the experimental data as well as those predicted with the original COSMO-RS is displayed in Table S5 and Figure 4. From Figure 4, it can be seen that the ARD between $\text{ln }X_{CO_{2}}^{COSMO-RS}$ and $\text{ln }X_{CO_{2}}^{Exp.}\;$is 78.2%, while it is only 10.3% between $\text{ln }X_{CO_{2}}^{corr.}$ and $\text{ln }X_{CO_{2}}^{Exp.}$, indicating that the corrected COSMO-RS is applicable for different kinds of DESs.

In summary, COSMO-RS is a predictive model, but the performance is not always satisfactory for the DESs with CO_2_. The model performance of CO_2_ solubility in DESs can be improved significantly with the corrected COSMO-RS.

### Further Investigation With COSMO-RS and Discussion

#### σ-Profiles Prediction

**Figure 5:**
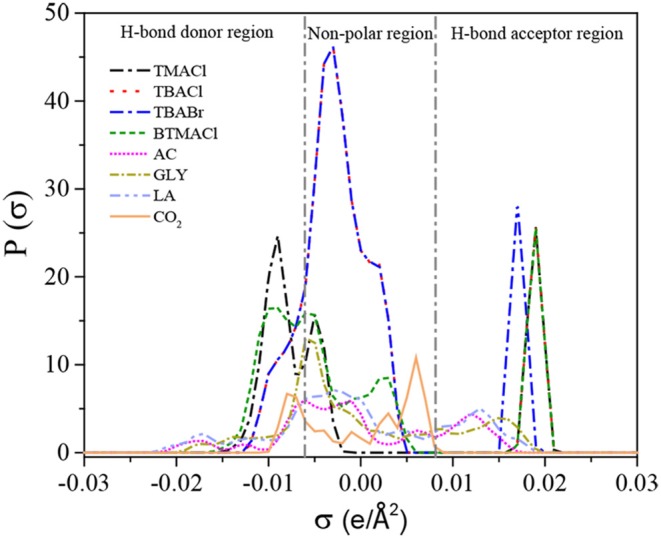
σ-profiles of HBAs, HBDs, and CO_2_.

The molecular interactions between two compounds can be linked to their σ-profiles, the wider the complementary of their σ-profiles (i.e., in the same region, the σ-profile for one compound increases, while that for the other compound decreases), the stronger the molecular interactions between these two compounds. In this work, in order to study the effects of HBAs and HBDs on the interactions with CO_2_, the HBAs of TMACl, TBACl, TBABr, and BTMACl as well as the HBDs of AC, GLY, and LA were selected to predict the σ-profiles. The predicted results are listed in Table S7 and illustrated in Figure 5. Following the previous work, the σ-profiles can be divided into three regions: H-bond donor region (σ < −0.0082 e/Å^2^), non-polar region (−0.0082 < σ < +0.0082 e/Å^2^), and H-bond acceptor region (σ > +0.0082 e/Å^2^) (Liu Y. R. et al., 2017). In the H-bond acceptor region, the σ-profiles of HBAs for TMACl and TBACl are almost the same. However, the σ-profile of TBACl has a wider region complemented with CO_2_ than that for TBACl in both H-bond donor and non-polar regions, indicating that TBACl has a strong interaction with CO_2_ compared to TMACl. Meanwhile, the location of the σ-profile peak in the H-bond acceptor region also reflects the interaction strength with CO_2_. TBACl and TBABr have the same σ-profile curves in the H-bond donor and non-polar regions, while in the H-bond acceptor region, the peak of TBACl (0.019 e/Å^2^) is located to the right of TBABr (0.017 e/Å^2^), evidencing that TBACl has a stronger interaction with CO_2_ than TBABr. The σ-profile prediction shows that both the alkyl chain length of cations and the different types of anions of HBAs can affect the CO_2_ solubility, agreeing with the experimental results of *X*_*CO*_2__ (TBACl-LA 1:2) > *X*_*CO*_2__ (TMACl-LA 1:2) (Zubeir et al., 2014) and *X*_*CO*_2__ (TBACl-AC 1:2) > *X*_*CO*_2__ (TBABr-AC 1:2) (Sarmad et al., 2017b) at the same temperature and pressure. Additionally, as shown in Figure 5, it can be seen that AC is more complementary with CO_2_ than GLY, and the σ-profile curve of AC is lower than that of GLY. This implies that AC as the HDB in DES has a strong interaction with CO_2_ with respect to GLY, being consistent with the experimental results of *X*_*CO*_2__ (BTMACl-AC 1:2) > *X*_*CO*_2__ (BTMACl-GLY 1:2) at the same temperature and pressure (Sarmad et al., 2017b). Therefore, the σ-profile can be used to reflect the interaction strength of a DES with CO_2_.

#### Excess Enthalpy Prediction

**Figure 6:**
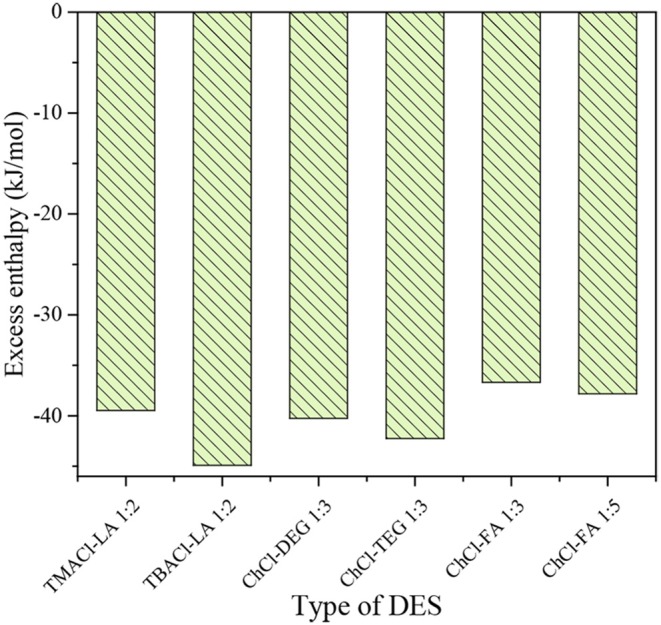
Excess enthalpy of DESs+CO_2_ system.

The total excess enthalpy of six kinds of DESs at infinitely dilute CO_2_ concentrations predicted by COSMO-RS was illustrated in Figure 6. These DESs can be divided into three types: (1) TMACl-LA 1:2 and TBACl-LA 1:2 have the same HBD and molar ratio but different HBA; (2) ChCl-DEG 1:3 and ChCl-TEG 1:3 have the same HBA and molar ratio but different HBD; (3) ChCl-FA 1:3 and ChCl-FA 1:5 have the same HBA and HBD but different molar ratios. The higher the absolute values of excess enthalpy, the stronger the interaction between DES and CO_2_, i.e., the higher the capacity for CO_2_ capture. As shown in Figure 6, the absolute excess enthalpy of TBACl-LA 1:2+CO_2_ is higher than that for TMACl-LA 1:2+CO_2_, indicating that TBACl-LA 1:2 has a higher capacity for CO_2_ capture. The comparison of ChCl-DEG 1:3+CO_2_ and ChCl-TEG 1:3+CO_2_ shows that ChCl-TEG 1:3+CO_2_ has a high absolute excess enthalpy compared to ChCl-DEG 1:3+CO_2_ system, being in agreement with the experimental results obtained by Sarmad et al. (2017b) and Ghaedi et al. (2017), i.e., increasing the alkyl chain length in HBA and HBD results in an increased CO_2_ solubility. Moreover, by increasing the molar ratio of HBD in DES, the absolute excess enthalpy of ChCl-FA 1:5+CO_2_ is increased compared to ChCl-FA 1:3+CO_2_ system, which agrees with the experimental results (Lu et al., 2015). Therefore, predicting excess enthalpy may be an efficient option for designing the potential DES for CO_2_ capture.

**Figure 7:**
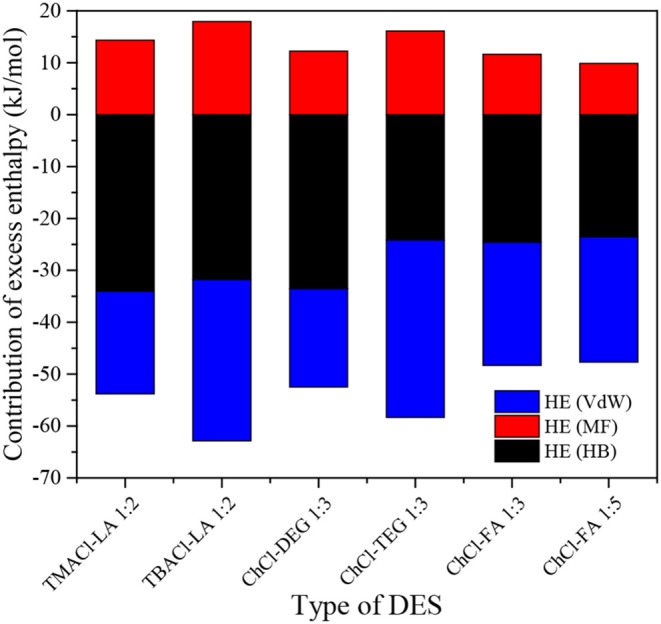
Contribution to the excess enthalpy of DESs+CO_2_ system.

Figure 7 displays the contribution of each interaction [H-bond (HB), misfit (MF), and Van der Waals force (VdW)] to the total excess enthalpy of DESs+CO_2_ systems. It is observable in Figure 7 that HB and VdW are consistently the dominant interactions for the DES+CO_2_ systems, followed by MF. Shukla et al. reported that the CO_2_ solubility in DES depends on the HB interactions between HBA and HBD (Shukla and Mikkola, 2018). Cao et al. indicated that the formation of HB between HBA and HBD enhanced the CO_2_ solubility in DESs (Cao et al., 2015). In addition, Atilhan et al. investigated the interactions between DES and SO_2_ by the quantum chemistry, confirming a dominant VdW interaction between DES and SO_2_ (Atilhan et al., 2019). These findings support our results.

## Conclusion

This work established a database containing 502 experimental data points for CO_2_ solubility and 132 for the Henry's constant of CO_2_ in DESs. This database was used for further verification and development of COSMO-RS.

The logarithmic CO_2_ solubility predicted with the original COSMO-RS shows ARDs of 62.2, 59.6, 63.0, and 59.1% for the DESs with the HBD:HBA at 1:2, 1:3, 1:4, and 1:5, respectively. The Henry's constants of CO_2_ in the DESs (1:1.5, 1:2, 1:3, 1:4, 1:5) predicted with the original COSMO-RS with ARDs of 13.7–36.3% are more accurate compared to the predicted CO_2_ solubilities. To improve the performance, COSMO-RS was corrected based on 502 data points of CO_2_ solubility in the DESs (1:2, 1:3, 1:4, 1:5). It shows that the adjustable parameters in the corrected COSMO-RS can be universal, the corrected COSMO-RS with the universal parameters can be used to reliably predict the CO_2_ solubility in DESs, and the ARDs for the logarithmic CO_2_ solubility in the DESs (1:2, 1:3, 1:4, 1:5) are of 6.8, 5.2, 6.6 and 4.7%, respectively. The corrected COSMO-RS with the universal parameters was further used to predict CO_2_ solubility in the DESs (1:6), showing that a much lower ARD (10.3%) compared to that with the original COSMO-RS (ARD, 78.2%). Additional, the σ-profiles can reflect the strength of molecular interactions between an HBA (or HBD) and CO_2_, and the dominant interactions for CO_2_ capture in DESs are the H-bond and Van der Waals force, followed by the misfit, according to the results of excess enthalpies.

This work provides a reliable tool for DESs screening and the corrected COSMO-RS can be used to quantitatively predict CO_2_ solubilities in DESs.

## Data Availability Statement

All datasets generated for this study are included in the article/Supplementary Material.

## Author Contributions

All authors listed have made a substantial, direct and intellectual contribution to the work, and approved it for publication.

### Conflict of Interest

The authors declare that the research was conducted in the absence of any commercial or financial relationships that could be construed as a potential conflict of interest.
